# Remifentanil or Alfentanil for Endotracheal Intubation

**DOI:** 10.5812/aapm.3465

**Published:** 2012-04-01

**Authors:** Federico Bilotta, Giovanni Rosa

**Affiliations:** 1 Department of Anesthesiology, Critical Care and Pain Medicine, Sapienza University of Rome, Rome, Italy

**Keywords:** Remifentanil, Alfentanil, Intubation, Intratracheal

Dear Editor, 

In their study Imani et al. (1) have compared the use of remifentanil (5 μg/kg) or alfentanil (50 μg /kg) as adjunct to propofol infusion (2 mg/kg) for rapid sequence anesthesia induction and tracheal intubation without the use of neuromuscular blocking agents in 100 American Socienty of Anesthesiologyists (ASA) I patients aged 20-50 years. In order to evaluate the effects on intubating conditions the ease of laryngoscopy, patency of vocal cords, jaw relaxation and limb movements were recorded at 1.5 minute after the start of anesthesia induction (10 seconds for opioid infusion + 45 seconds for distribution + 5 seconds for propofol injection + 30 seconds for distribution). According their results both remifentanil and alfentanil provided good intubating conditions, the use of remifentanil was associated to higher rate of vocal cords patency.

This study provides an interesting and clinically relevant contribution toward a debated arena (2, 3). Some elements need to be highlighted: the mean age of enrolled patients is relatively low (range 20-50, mean 35 and 32 years), anesthesia induction related desaturation and hemodynamic conditions (including changes in heart rate and blood pressure) were not recorded.

In young patients the associated co-morbidity is generally low and in this study only patients with ASA physical status I were selectively recruited. This might limit the reproducibility in patients with associated cardiac, vascular and metabolic disease that might present with slower distribution time (4, 5).

In order to evaluate the usefulness and clinical characteristics of a rapid sequence anesthesia induction approach is important to record the effects on spontaneous ventilation, episodes of apnea and desaturation (6-8). These variables describe a relevant risk associated to a rapid induction sequence technique and are quality indicators.

Remifentanil used for anesthesia induction has prominent hemodynamic effects including marked bradycardia and arterial hypotension (9, 10). These hemodynamic effects are common, and possibly additive, to the effects exerted by propofol injection when it is administered at an high dose/time rate (4). In patients with coronary artery disease or chronic arterial hypertension these effects can markedly reduce organ perfusion and might lead to temporary ischemia.

In conclusion, the results of the study from Imani et al. (1) provide evidence for a useful and effective clinical approach to rapid sequence anesthesia induction, without the use of neuromuscular blocking, in young patients that present no associated co-morbidities. The reproducibility in specific subgroups of patients at high risk for difficult intubation, including obese and parturient, might further extend the clinical indication for this approach.
